# Supplementary material for: Content-rich biological network constructed by mining PubMed abstracts
Source: BMC Bioinformatics. 2004 Oct 8;5:147. doi: 10.1186/1471-2105-5-147 (PMC528731; doi:10.1186/1471-2105-5-147)
Supplement: Additional File 5 — The original Chilibot query results of the term "long-term potentiation (LTP)" and 22 other terms, limiting the latest references analyzed to the years 1990, 1995, 2000, and 2004. [file 1471-2105-5-147-S5.bz2 › chilibotAdditionalFile5/ltp2000/html/TAU_KV4.2.html]

 


 **TAU** and **KV4.2** 
  
Found 2 abstracts in PubMed,  **2 abstracts were retrieved and analyzed**.  


---

 Search Google  |
 PDF files only 
|  EDU domain only 

---

- J Physiol, 1997   **Effects of development and thyroid hormone on K currents and K channel gene expression in rat ventricle.** .
  1.
  In rat heart, three K channel genes that encode inactivating transient outward ITO like currents are expressed.
  During development the predominant K channel mRNA species switches from Kv1.4 to **Kv4.2** and Kv4.3.
  However, no functional correlate of this isoform switch has been reported.
  We investigated action potential characteristics and ITO in cultured neonatal rat ventricular myocytes and adult rat hearts.
  We further examined whether the changes in K channel gene expression and the associated electrophysiology that occurs during development could be induced by thyroid hormone.
  2.
  In myocytes isolated from right ventricle of adult rat heart, action potential duration was short and independent of rate of stimulation.
  The density of ITO was 21.5 1.8 pA pF 1 n = 21 .
  Recovery from inactivation was best described by a single exponential **tau** fast = 31.7 2.7 ms, n = 13 .
  The current remaining at the end of a 500 ms pulse ISUS was 6.2 0.5 pA pF 1 n = 19 .
  3.
  In contrast to adult cells, action potential duration was prolonged and was markedly rate dependent in cultured neonatal rat ventricular myocytes.
  The current density of ITO measured in cultured ventricular myocytes from 1 to 2 day old rats was 10.1 1.5 pA pF 1 n = 17 .
  The recovery from inactivation for ITO was best described by the sum of two exponentials **tau** fast = 64.3 8.8 ms, 54.4 10.2%.
  **tau** slow = 8216 2396 ms, 37.4 7.9%.
  n = 5 .
  ISUS was 4.4 0.6 pA pF 1 n = 17 .
  Steady state activation and inactivation were similar in adult and neonatal ventricular myocytes.
  4.
  In neonatal myocytes treated with thyroid hormone, tri iodothyronine T 00 nM , action potential duration was abbreviated and independent of stimulation rate.
  Whilst T3 did not significantly increase ITO density 24.0 2.9 pA pF 1.
  n = 21 in T3 treated cells cf.
  20.1 3.0 pA pF 1.
  n = 37 in untreated controls , the recovery from inactivation of ITO was accelerated **tau** fast = 39.2 3.6 ms, 82.2 8.9%, n = 9 .
  T3 did however, increase ISUS current density 4.7 0.77 pA pF 1.
  n = 37 and 7.0 0.7 pA pF 1, n = 21, in control and T3 treated cells, respectively.
  5.
  The effects of T3 100 nM were associated with a marked decrease in the expression of Kv1.4 at the mRNA and protein level, and an increase in the expression of Kv4.3 without changes in **Kv4.2** mRNA levels.
  6.
  The findings of the present study indicate that postnatal development involves a shortening of action potential duration and an increase in the density of ITO.
  Furthermore, we show that development is also associated with a loss of action potential rate dependence, and an acceleration in the rate of recovery of ITO.
  We propose that these functional effects occur as a consequence of the previously reported developmental Kv1.4 to **Kv4.2** Kv4.3 isoform switch.
  In cultured neonatal myocytes, T3 induced many of the electrophysiological and molecular changes that normally occur during postnatal development, suggesting that this hormone may play an important role in postnatal electrophysiological development.

  - Cardiovasc Res, 1997   **Electrophysiological and pharmacological correspondence between **Kv4.2** current and rat cardiac transient outward current.** .
    OBJECTIVE The transient outward current ITO plays an important role in early repolarization and overall time course of the cardiac action potential.
    At least two K channel alpha subunits cloned from cardiac tissue Kv1.4 and **Kv4.2** encode rapidly inactivating channels.
    The goal of this study was to determine functional and pharmacological properties of **Kv4.2** expressed in mammalian cells, especially those that would differentiate between both isoforms in comparison to native ITO.
    METHODS Both **Kv4.2** and Kv1.4 isoforms were stably expressed in mouse L cell lines, and expressed currents were studied using whole cell voltage clamp techniques.
    RESULTS The expressed **Kv4.2** currents displayed fast inactivation with a half inactivation potential of 41 mV.
    Recovery from inactivation was rapid **tau** recov = 160 ms at 90 mV and strongly voltage dependent.
    Flecainide 10 microM had minimal effects on Kv1.4 currents, but reduced **Kv4.2** peak current by 53% and increased the apparent rate of inactivation consistent with open channel block.
    Quinidine 10 20 microM reduced the peak current and accelerated the apparent rate of inactivation in both isoforms.
    The **Kv4.2** current displayed use dependent unblock in the presence of 4 AP.
    CONCLUSIONS The functional properties of **Kv4.2**, especially the flecainide sensitivity, resemble those of ITO in rat and human myocytes better than those of Kv1.4.
    These results provide the necessary functional support for the hypothesis that **Kv4.2** is a major isoform contributing to cardiac ITO, consistent with independent biochemical and molecular evidence that indicates that **Kv4.2** is readily detected in rat myocytes.
